# Potential role of serum copeptin among smoker T2DM patients with emphasis to ACE I/D gene polymorphism predicting DN

**DOI:** 10.1038/s41598-024-62865-8

**Published:** 2024-06-06

**Authors:** Mona Mohamed Taha, Mohamed Ahmed Yehia Zakaria, Yasmine Hamdy Eisa, Maysa Hatem Rashed

**Affiliations:** 1 Department of Environmental and Occupational Medicine, National Research Centre, Buhouth Street, Dokki, Cairo, Egypt; 2 Department of Forensic Medicine and Clinical Toxicology, Faculty of Medicine, October 6 University, Giza, Egypt; 3 Department of Public Health and Community Medicine, Faculty of Medicine, October 6 University, Giza, Egypt; 4 Department of Biochemistry and Molecular Biology, Faculty of Medicine, October 6 University, Giza, Egypt

**Keywords:** DN, Copeptin, KIM-1, Kidney impairment, T2DM, Urinary Pb, Mutation, Biochemistry, Genetics

## Abstract

Diabetic nephropathy represents one of the main long-term complications in T2DM patients. Cigarette smoking represents one of modifiable renal risk factors to kidney damage due to lead (Pb) exposure in these patients. Our goal is to investigate serum copeptin and Kidney injury molecule-1 (KIM-1) and urinary lead (UPb) in type 2 diabetes mellitus (T2DM) patients even smokers and non-smokers groups and compared to corresponding health controls and assess its associations with Angiotensin-Converting enzyme Insertion/Deletion polymorphism [ACE (I/D)] polymorphism in diabetic nephropathy progression in those patients. In present study, 106 T2DM patients and 102 healthy control individuals were enrolled. Serum glucose, copeptin, KIM-1, total cholesterol (TChol), triglycerides (TG), estimated glomerular filtration rate (eGFR) and UPb levels and ACE (I/D) polymorphisms were assessed in both groups. Results mentioned to significant variations in all parameters compared to in T2DM group compared to control group. Serum copeptin and UPb demonstrated significant difference in diabetic smokers (DS) and diabetic non-smokers (DNS) groups while KIM-1 exhibited significant change between DNS and healthy control non-smokers (CNS) groups. Positive relation was recorded between serum glucose and KIM-1 while negative one was found between serum copeptin and TChol. D allele was associated with significant variation in most parameters in T2DM, especially insertion/deletion (ID) polymorphism. ROC curve analysis (AUC) for serum copeptin was 0.8, p < 0.044 and for Kim-1 was 0.54, p = 0.13 while for uPb was 0.71, p < 0.033. Serum copeptin and UPb might be a prognostic biomarker for renal function decline in smoker T2DM patients while KIM-1 was potent marker in non-smoker T2DM with association with D allele of ACE I/D gene polymorphism.

## Introduction

Diabetes mellitus, in terms of hyperglycemia, represents a metabolic disorder caused by disturbances in insulin production, action, or both^1^. Diabetic nephropathy (DN) affects about 40% of diabetic patients, whether with type 1 or type 2 diabetes^2^.

Copeptin, a 39-amino acid glycopeptide, is secreted in equal amounts with arginine-vasopressin (AVP) in the posterior pituitary from the AVP preprohormone. Recent study reported an association between copeptin levels and increased risk of developing chronic kidney disease in the general population^3^. Also, renal dysfunction, or DN major complications, was characterized by a progressive rise in albuminuria that was followed by a fall in glomerular filtration rate (GFR)^4^.

Kidney injury molecule-1 (KIM-1), which represents a cellular receptor that is located on T- lymphocytes and dendritic cells, is shown to be involved in both autoimmune conditions and responses. In both acute and chronic kidney injury, upregulation of its levels is markedly recorded in the damaged proximal tubule cells^5^. KIM-1 has the ability to oxidize lipoproteins in addition to its involvement in identifying apoptotic cell surface, phosphatidylserine and epitopes. Dead and necrotic debris engulfment in the damaged epithelial tubules is interceded by KIM-1. It can escape the unwanted attachment of exfoliated cells to fibronectin and reduce cast development and tubular obstruction. The preceding characteristic may suggest KIM-1 to be an ideal biomarker of kidney injury^6^.

Substantial clinical evidence indicates that smoking has a deleterious and negative impact on both kidney dimensions and renal function, as well as CKD progression and/or development from many etiologies, e.g., diabetes mellitus and hypertension. So, cigarette smoking represents one of the most significant and important risk factors for modifying renal functions^7^. Furthermore, lead (Pb) is a hazardous non-essential trace metal with high toxicity to human health. Inhalation of cigarette smoke and Pb-contaminated ambient air may also contribute to its exposure in the general population, in addition to its exposure routes mainly through diet, drinking water, and dust^8^. Additionally, Hagedoorn et al.^9^ concluded that T2DM patients may be more susceptible to renal dysfunction because of Pb exposure compared with the general population, or that the Pb nephrotoxicity mechanism in T2DM is different from that in non-diabetic individuals.

Mahwish et al.^10^ mentioned the association between DN, dyslipidemia, and ACE I/D genotypes. The gene for ACE is located on chromosome 17q23. Three genotypes of ACE were identified: DD homozygote, ID heterozygote, and II homozygote^11^. An insertion (I allele) and deletion (D allele) functional polymorphism of the ACE I/D gene was revealed by another study. According to Lakkakula et al.^12^, the D allele was associated with high circulating ACE levels in addition to the relationship between DN and ACE I/D polymorphism. Mahwish et al.^10^ studied the insertion/deletion polymorphism with renal and cardiovascular complications in T2DM. Another study found a relationship between the I/D polymorphism of ACE and ESRD due to diabetes^11^.

Our goal is to investigate serum copeptin and Kidney injury molecule-1 (KIM-1) and urinary lead (UPb) in type 2 diabetes mellitus (T2DM) patients even smokers and non-smokers groups and compared to corresponding health controls and assess its associations with Angiotensin-Converting enzyme Insertion/Deletion polymorphism [ACE (I/D)] polymorphism in diabetic nephropathy progression in those patients.

## Results

Table 1 revealed that only diastolic pressure demonstrated difference between diabetic patients and healthy control group, while it is of no significance as diastolic pressure mean is still within normal range. In diabetic patients, 51.9% were smokers (DS) and 48.1% non-smokers (DNS) while 49% in healthy control were smokers (CS) and 51% of non-smokers (CNS).

**Table 1:** General characteristic data of participants.

Parameters	Diabetic patients (n = 106)	Healthy Control (n = 102)	Test of significance	P value
Age (years)	51.61 ± 6.18	50.27 ± 5.98	1.59^a^	0.11
Smoking habits				
Yes	55 (51.9%)	50 (49%)	0.17^b^	0.39
No	51 (48.1%)	52 (51%)		
Duration of smoking (Years)	20.27 ± 11.61	17.84 ± 9.8	1.0^a^	0.32
Diastolic pressure (mmHg)	85.29 ± 9.45	79.17 ± 9.42	3.24^a^	0.002
Systolic pressure (mmHg)	129.46 ± 16.51	127.104 ± 9.54	0.67^a^	0.5

^a^t test.

^b^χ^2^.

Table 2 recorded high significant increase in serum glucose, HbA_1_C (%), serum KIM-1, copeptin, cholesterol, TG and urinary Pb levels with significant decrease in eGFR in T2DM patients.

**Table 2:** Biochemical parameters levels in diabetic patients and healthy control subjects.

Parameters	Diabetic patients (n = 106)	Healthy Control (n = 102)	t Test	P value
Fasting s. glucose (mg/dL	165.11 ± 84.76	90.37 ± 20.80	8.66	< 0.001
HbA_1_C (%)	7.34 ± 1.46	5.26 ± 0.71	10.45	< 0.001
Serum KIM-1 (ng/mL)	30.29 ± 15.26	24.78 ± 8.73	2.95	< 0.004
Serum Copeptin (ng/mL)	567.10 ± 125.45	472.74 ± 102.38	5.54	< 0.001
Serum Chol (mg/dL)	179.37 ± 46.85	132.97 ± 42.32	7.14	< 0.001
Serum TG (mg/dL)	197.10 ± 76.60	137.48 ± 66.87	5.71	< 0.001
uPb (ng/mg creat)	0.34 ± 0.44	0.19 ± 0.14	2.66	< 0.009
eGFR (mL/min/1.73m^2^)	120.13 ± 40.67	132.57 ± 25.83	2.5	< 0.012

**Figure 1:**
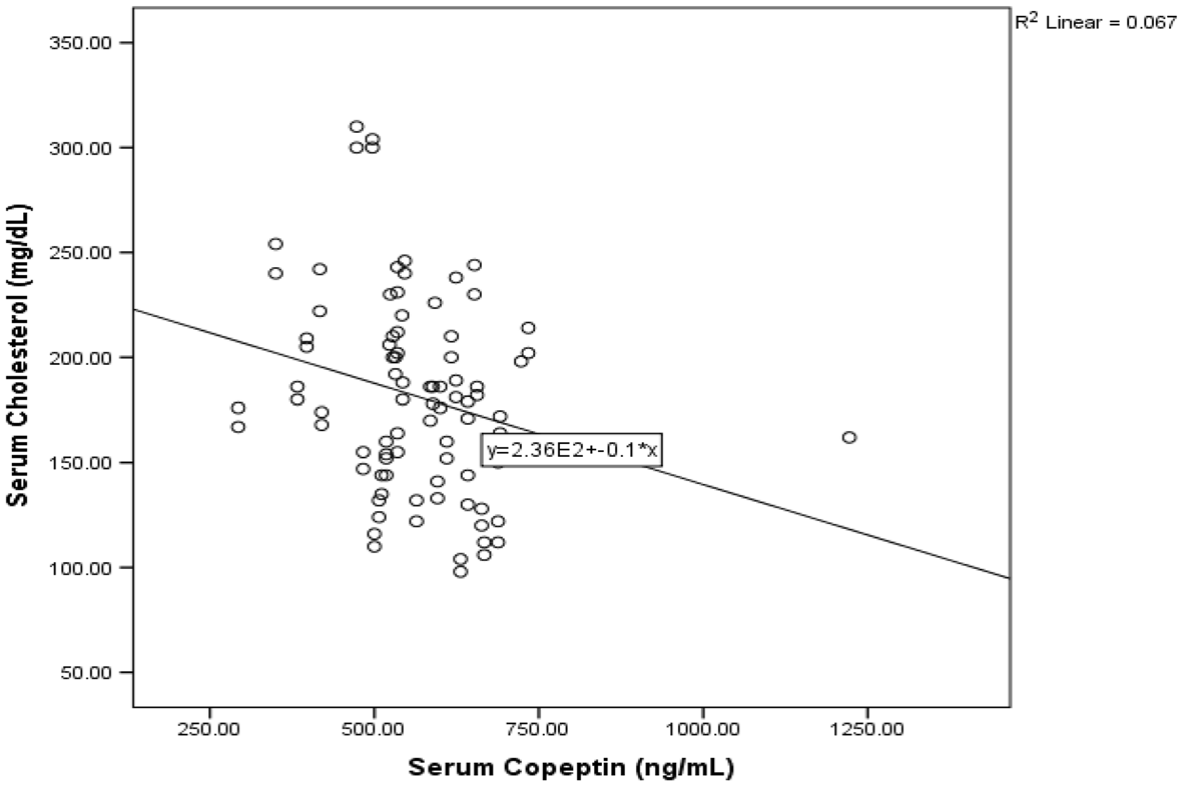
Correlation between serum glucose and serum Kim-1 in diabetic patients.

**Figure 2:**
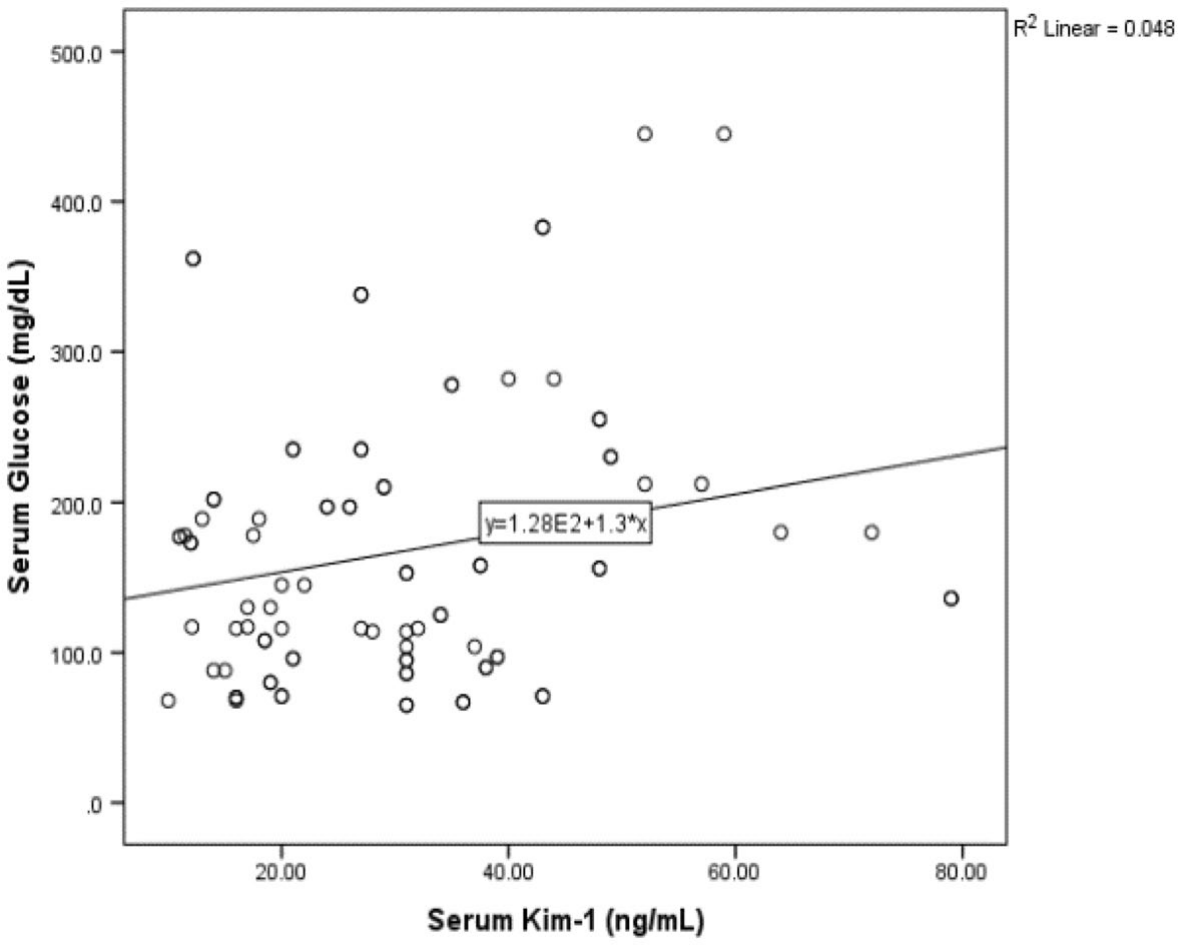
Correlation between serum cholesterol and serum copeptin in diabetic patients.

Figure 1 showed positive correlation between serum glucose and serum KIM-1 among diabetic patients (r = 0.22, P < 0.023), while negative significant correlation was shown between serum cholesterol and serum copeptin among those diabetic patients in Fig. 2 (r = − 0.26, P < 0.015).

Table 3 demonstrated high significant variations in serum glucose, HbA_1_C (%), serum KIM-1, copeptin, cholesterol and TG between T2DM patients and healthy control either smoker and non- smoker subjects. In addition, serum KIM-1 illustrated significant difference between DS and CNS groups. Also, serum copeptin revealed significant change between DS and either DNS and CNS groups. Urinary Pb showed significant change in CNS and both DS or CS groups. Finally, eGFR exhibited significant change in CNS group and DS group.

**Table 3:** Biochemical parameters levels in diabetic patients and healthy control subjects (smoker and non-smoker).

Parameters	Smokers		Non-smokers		Anova	P value
	DS (n = 55)	CS (n = 50)	DNS (n = 51)	CNS(n = 52)		
Fasting s. glucose (mg/dL)	169.10 ± 47.64	88.30 ± 14.69^a^	160.86 ± 74.70^b^	92.37 ± 16.20^a^	24.23	< 0.001
HbA_1_C (%)	7.49 ± 1.48	5.47 ± 0.39^a^	7.16 ± 1.42^b^	5.62 ± 0.69^a^	37.5	< 0.001
Serum KIM-1 (ng/mL)	29.21 ± 12.28	28.04 ± 7.43	31.64 ± 16.52	20.29 ± 6.51^a^	7.8	< 0.001
Serum Copeptin (ng/mL)	543.20 ± 114.83	478.12 ± 110.4^a^	596.24 ± 132.9^a^	466.32 ± 92.79^a^	11.86	< 0.001
Serum Chol (mg/dL)	178.57 ± 42.10	127.95 ± 28.70^a^	180.24 ± 51.93^b^	137.87 ± 52.22^a,b^	16.9	< 0.001
Serum TG (mg/mL)	201.51 ± 75.85	132.61 ± 67.99^a,^	192.29 ± 76.78	142.24 ± 66.18^b^	10.39	< 0.001
uPb (ng/mg creat)	0.41 ± 0.38	0.28 ± 0.13	0.25 ± 0.47^a^	0.11 ± 0.098^a,b^	5.4	< 0.002
eGFR (mL/min/1.73m^2^)	120.55 ± 41.32	130.02 ± 26.82	119.65 ± 40.36	135.24 ± 24.77^a^	2.5	0.059

^a^Significant from DS at P < 0.001.

^b^Significant from CS at P < 0.001.

**Figure 3:**
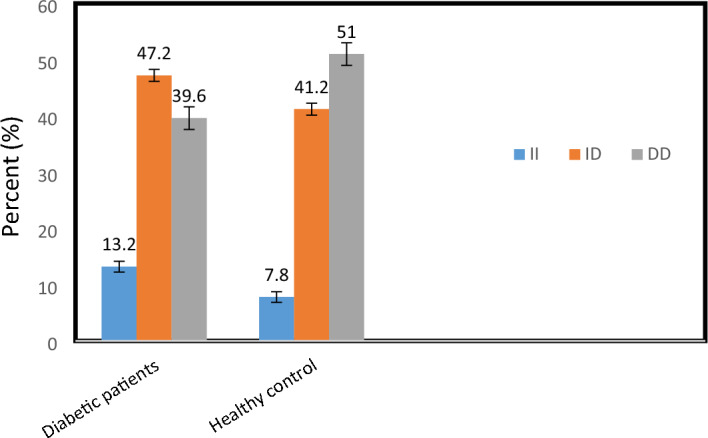
Genotype frequency of ACE (I/D) genotypes in diabetic patients and healthy control subjects.

Figure 3 showed non-significant difference (χ^2^ = 3.32, P = 0.19) in distribution frequency of ACE (I/D) polymorphisms between diabetic patients and healthy control. The ID polymorphism was the dominant one with 47.2% in diabetic patients while the DD polymorphism represented 39.6% and II polymorphism reported lower frequency in both groups.

Table 4 found significant difference in serum glucose, cholesterol and HbA_1_C (%) levels in different ACE (I/D) polymorphisms between diabetic patients and healthy control. Serum KIM-1, copeptin, TG and eGFR showed significant difference in either DD and ID polymorphism between diabetic patients and healthy control. Serum cholesterol exhibited high significant difference in II genotypes and urinary Pb levels showed significant rise only in ID genotypes in diabetic patients.

**Table 4:** Biochemical parameters levels in different ACE I/D genotypes in diabetic patient and normal control groups.

Parameters	DD		ID		II	
	Diabetic patient n = 42)	Normal control (n = 52)	Diabetic patient (n = 50)	Normal control (n = 42)	Diabetic patient (n = 14)	Normal control (n = 8)
Fasting s. glucose (mg/dL	167.57 ± 97.00	93.25 ± 19.85	164.00 ± 77.10	85.48 ± 22.37	161.71 ± 76.85	97.38 ± 13.46
P value	< 0.001		< 0.001		< 0.031	
HbA_1_C (%)	7.09 ± 1.16	5.22 ± 0.73	7.66 ± 1.68	5.28 ± 0.60	6.87 ± 1.18	5.21 ± 0.59
P value	< 0.001		< 0.001		< 0.017	
Serum KIM-1 (ng/mL)	30.86 ± 16.39	24.81 ± 8.54	30.76 ± 14.29	24.25 ± 8.18	27.71 ± 8.2	24.85 ± 7.08
P value	< 0.023		< 0.011		0.42	
Serum Copeptin (ng/mL)	599.84 ± 154.4	497.73 ± 109.51	541.46 ± 98.84	447.58 ± 85.07	549.71 ± 84.28	448.71 ± 116.43
P value	< 0.001		< 0.001		0.07	
Serum Chol (mg/dL)	181.0 ± 48.0	134.0 ± 42.0	175.83 ± 43.37	136.50 ± 45.04	186.0 ± 56.36	108.14 ± 19.51
P value	< 0.001		< 0.001		< 0.002	
Serum TG (mg/mL)	190.95 ± 68.69	124.91 ± 59.17	191.70 ± 60.57	151.31 ± 73.51	233.14 ± 125.46	149.0 ± 72.81
P value	< 0.001		< 0.01		0.12	
uPb (ng/mg creat)	0.23 ± 0.30	0.20 ± 0.14	0.41 ± 0.53	0.17 ± 0.15	0.32 ± 0.15	0.22 ± 0.11
P value	0.59		< 0.032		0.21	
eGFR (mL/min/1.73m^2^)	115.53 ± 45.30	131.50 ± 24.17	118.78 ± 36.59	135.45 ± 27.67	137.64 ± 37.93	122.0 ± 27.57
P value	< 0.038		< 0.019		0.31	

**Figure 4:**
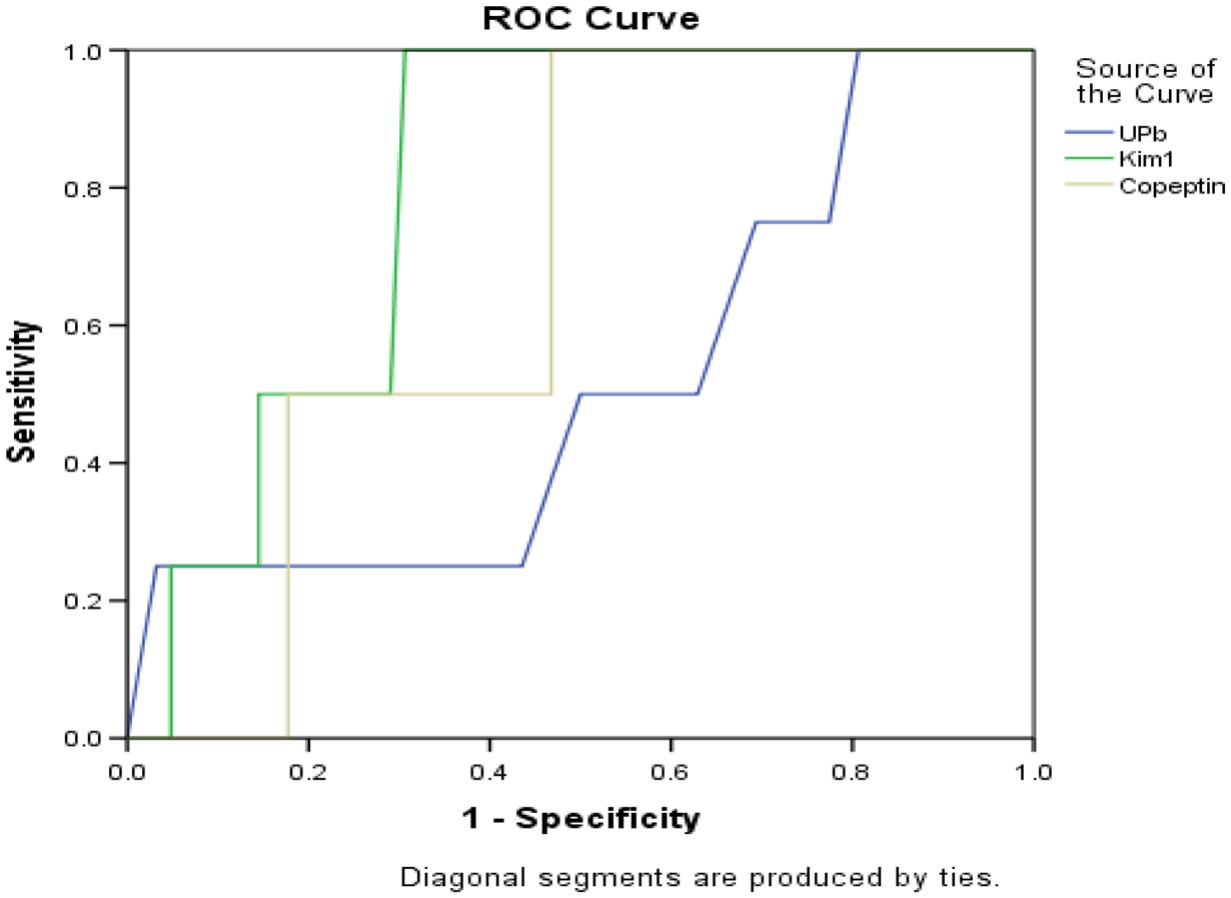
ROC curve analysis of serum copeptin, KIM-1 and urinary Pb for early prediction of DN in smoker T2DM.

Figure 4 illustrated that crude area under the ROC curve (AUC) of serum copeptin was 0.8, p < 0.044 with a cut off value of 461.2 ng/mL which denoted 91.5% sensitivity and 82.3% specificity. As regard serum KIM-1, crude area under ROC curve (AUC) was 0.54, p = 0.13 with a cut off value of 25.6 ng/mL which denoted 84.7% sensitivity and 77.4% specificity. Regarding UPb, AUC of uPb was 0.71, p < 0.033 with a best of 0.17 ng/mg creat and sensitivity of 88.5% and sensitivity of 81.2% specificity. That suggesting that serum copeptin and uPb level are more sensitive than serum Kim-1 as early marker for renal impairment in smoker T2DM.

## Discussion

Cigarette smoking was revealed to be associated with increased risk of progression in chronic kidney disease as well as kidney failure in either diabetic patient and/or those affected by hypertension^20^.

Pathogenesis of DN is multifactorial and contribution of different proteins, genes and environmental factors was seen to the onset of the disease^21^.

Current study recorded that 51.9% of diabetic patients were smokers (DS) compared to 49% in healthy control were smokers (CS) and 48.1% in diabetic subjects were non-smokers (DNS) and 51% of non-smokers in healthy control one (CNS). Only significant difference in diastolic pressure between diabetic and healthy control individuals while the diastolic pressure was in the normal range. Recent study revealed that in DN there was a seasonal variation of blood pressure that increase in those patients with a difference in between peak and nadir values is 9.03/5.08 mmHg in DN patients^22^.

We identified serum copeptin, KIM-1 and urinary Pb levels with eGFR as well as ACE I/D gene polymorphism that could affect DN progression in T2DM either smokers and non-smokers individuals.

A rise in KIM-1 levels in T2DM patients were recorded in our study. In line with our study Guo et al.^23^ mentioned that hyperglycemia leads to elevation in KIM-1 level, indicating high glucose-induced damage to renal tubular cells. The latter finding can explain the positive correlation between serum glucose and KIM-1 levels among diabetes patients by controlling cell autophagy and apoptosis, KIM-1 could cause damage and healing of renal tubular epithelial cells which attributed to hyperglycemia. Also, high serum copeptin levels with decline in eGFR in T2DM patients was compatible with Noor et al.^4^ results who found significant association of copeptin with decline in eGFR. Wiromrat et al.^24^ concluded that hyperglycemia may contribute to increased copeptin possibly through inducing hypovolemia via glucosuria or by increasing plasma osmolality.

In line with our study, Velho et al.^25^ investigated individuals with type 1 or type 2 diabetes as well as in the healthy controls and they found that plasma copeptin was linked to perturbations in eGFR, as well as to the impairment in kidney function and the progression of DN during follow-up in both cohorts. Other study conducted by Andrii et al.^26^ demonstrated that DN patients with high serum levels of copeptin were shown to have more serious violations of glucose metabolism in comparison to patients with normal eGFR.

Current study recorded significant rise in urinary Pb in T2DM patients which is in line with Zhou et al.^27^ who reported more nephrotoxic effects of lead (Pb) nitrate in experimental diabetic rats that is following oxidative stress in diabetic rats compared to non-diabetic one. Type 2 diabetes and chronic kidney disease are separately linked to environmental exposure to lead (Pb). In a study focused on determining how concurrent exposure to this hazardous metal affected the probability of developing diabetes and renal damage conducted by Yimthiang et al.^28^. The exposure levels to lead had been determined to be low to moderate and considered in tandem, their results indicated that kidney function and the risk of diabetes are equally impacted by the exposure profiles to lead.

Negative relation was mentioned between serum cholesterol and serum copeptin, Korkmaz et al.^29^ suggested that copeptin levels may be affected by stress.

Current study demonstrated high significant variations in serum KIM-1, copeptin and urinary Pb levels between smokers and non-smokers groups in T2DM patients and healthy control groups. Tascon et al.^30^ suggested renal injury (that was related to smoking) shares pathophysiological mechanisms with DN and hypertension. Other study proposed that cigarette smoking impacts on kidney may be resulted through vascular endothelial cell damage which is a consequence of increasing oxidative stress and leukocytes activation which could lead to an increase in inflammatory cytokines release^20^.

We found that serum copeptin and urinary Pb recorded significant difference between DS and CNS groups. Henrique et al.^31^ declared that repeated dehydration and hyperosmolarity which is due to long-term hyperglycemia in diabetic patients can result chronic kidney damage through serum copeptin secretion and AVP affection. High levels of urinary Pb resulting lead nephrotoxicity have been suggested to that probably Pb enters renal proximal tubule cells, by endocytosis after low-molecular-weight proteins binding, where inhibition in renal mitochondrial respiratory function may result due to Pb toxicity, leading to oxidative stress through reactive oxidative species formation, intracellular depletion of glutathione levels and apoptosis^32^.

Serum KIM-1 recorded significant difference in DS group and CNS group indicating that KIM-1 could be an ideal kidney damage biomarker in T2DM either smoker or non-smokers and control individuals. Balu et al.^33^ demonstrated that KIM-1 is markedly up regulated and inserted into the proximal tubules' apical membranes where it remains in the epithelial cells until full cell recovery.

Our results illustrated that urinary Pb showed significant change in CNS and both DS or CS groups and eGFR exhibited significant change in CNS group and DS group. Nakhaee et al.^34^ suggested that chronic lead poisoning can cause chronic kidney disease with reducing in renal glomeruli number and volume. Orth et al.^35^ confirmed that cigarette smoking causes decline in eGFR that is proteinuria independent and this decline increased with increasing cigarette smoking exposure.

Present study recorded that in T2DM patients the ID genotypes was the most frequent one followed by DD and II of ACE genotypes and that results was in line with Jayapalan et al.^36^ who claimed that ID genotype significantly raised the risk of the DN. In meta-analysis conducted by Lakkakula et al.^12^, they confirmed that the D allele of the ACE I/D polymorphism was a susceptible factor to DN. Moreover, Hamad et al.^37^ demonstrated that development of DN is strongly related with the ACE I/D gene polymorphism, indicating that people with DD genotype are more likely to cause DN, but they also produce greater amount of ACE (Fig. 3).

Our findings illustrated association of DD and ID genotypes with significant variations in most parameters in T2DM patients while urinary Pb levels showed significant rise only in ID genotype. That mentioned to the association of D allele with tubular damage. The D allele was shown to behave as a recessive trait that required the presence of two alleles for contributing in ESRF progression^38^. The D allele of the ACE I/D gene is associated with high enzyme activity and was associated with kidney damage and advancement of CKD in DN^39^.

In the current study, we found that serum KIM-1 showed significant difference in DD genotype and ID polymorphisms among diabetic patients and healthy controls. Zeng et al.^40^ studied variations among diabetic patients in their susceptibility to DN regarding ACE I/D polymorphisms. They demonstrated that in Asian and Chinese populations as well as type 2 diabetics, there is a correlation between the ACE I/D gene polymorphism and the risk of DN. The DD genotype and the ACE D allele are risk factors for DN. On the other hand, the ACE II genotype appears to be a DKD protective factor. Aljorani et al.^41^ conducted a study to evaluate biomarkers including KIM-1 to help in early prediction of DN among type 2 diabetic patients, they showed a significant increase in serum and urinary levels of KIM-1 and its negative correlation with eGFR which was highly suggestive that KIM-1 could be sensitive and specific biomarker of DN among type 2 diabetic patients.

Our study illustrated that diagnostic value of serum copeptin for identification of DN by using the ROC curve analysis where the best cut off value was 461.2 ng/mL with 91.5% sensitivity and 82.3% specificity and for UPb, ROC curve analysis indicated the best cut off value was 0.23 ng/mg creat with sensitivity of 88.5% and sensitivity of 81.2% specificity (Fig. 4). Our results was in agreements with El-Soudany et al.^42^ who recorded that an increase in plasma copeptin level could be a good indicator for renal function deterioration in diabetic patients. This suggested that serum copeptin and uPb could be used to monitor smoker diabetic at risk for developing DN.

## Conclusion

The contribution of serum copeptin may be a promising marker in combination with UPb for renal outcome prediction as it results in renal functions worsening or kidney impairment especially in diabetic smokers’ individuals compared with non-smokers diabetic ones and its association with nephropathy. Furthermore, we can conclude that serum copeptin is better than serum KIM-1 in diabetic smokers’ T2DM. In addition, this study supports the view of association between the D allele of ACE gene, especially ID polymorphism and raised ESRD and/or DN risk in Egyptian T2DM patients.

Limitation of our study was the limited number of included subjects. Further prospective studies with a larger sample are needed to address these issues.

## Subjects and methods

In our case–control study, 208 people in total were enrolled. Two groups of participants were formed. 102 healthy controls and 106 T2DM patients from the Kasr Al-Aini hospital's diabetes clinic formed up group I. Diabetes had an average duration of 15.6 + 5.46 years. Age, socioeconomic status, and smoking habits were the same for the diabetes patients and the healthy controls. All subjects provided a written informed consent for their involvement in the study that is performed in accordance with the Declaration of Helsinki. A questionnaire with questions about demographics, clinical history, and environmental history was completed. Blood samples were collected from each participants after a 12-h fast glycated haemoglobin (HbA_1_c), serum creatinine, KIM-1, and copeptin levels analysis in addition to genetic analysis of ACE I/D gene. Urine sample with no preservative was collected to measure creatinine and urinary lead levels. Any participants with a history of renal dysfunction would be excluded.

### Methods

#### Biochemical analysis

##### Blood glucose

Analysis of fasting blood glucose was done according to Trinder^13^ colorimetric method.

##### Glycated hemoglobin (HbA_1_c).

HbA_1_c (%) was assessed according to Bates^14^ by ion exchange method.

##### Determination of serum cholesterol

Total cholesterol (TC) in serum was determined by Richmond^15^ colorimetric methods.

##### Determination of serum TG

Total triglyceride (TG) in serum was performed according to Fassati and Prencipe methods^16^ through colorimetric method.

##### Determination of serum Copeptin

Serum copeptin level was measured through an enzyme-linked immunosorbent assay (ELISA) commercial kit (Fine test co,Ltd).

##### Measurement of serum KIM-1

Serum KIM-1 levels was achieved using an (ELISA) commercial kit (SinoGeneClon Biotech Co., Ltd).

##### Measurement of serum and urinary creatinine

Serum and urinary creatinine (Cr) levels were carried out through Jaffe kinetic method according to Bartels^17^ without deproteinization. Urine creatinine was measured for adjusting urinary lead values.

##### Calculation of estimated glomerular filtration rate (eGFR)

Calculation of estimated GFR was according to Inker et al.^18^ using Chronic Kidney Disease-Epidemiology Collaboration (CKD-EPI) equation.

##### Measurement of urinary Pb levels by Heavy metals measured by ICP mass in urine

Thawing of urine samples were done slowly and diluted 1/5 with 0.5% HNO_3_. ^103^Rh was added to each sample at a final concentration of 1 µg/L as internal standard and thereafter analysed. ICP-sf-MS instrument (An ELEMENT 2) (Thermo Scientific, Bremen, Germany) was used for determination of ^208^Pb in low-resolution mode. Sample introduction was acieved using an ESI-Fast-system (Elemental Scientific, Mainz, Germany) connected to a Micromist nebuliser with a cyclon spray chamber.

##### Genetic determination of ACE I/D

**Figure 5:**
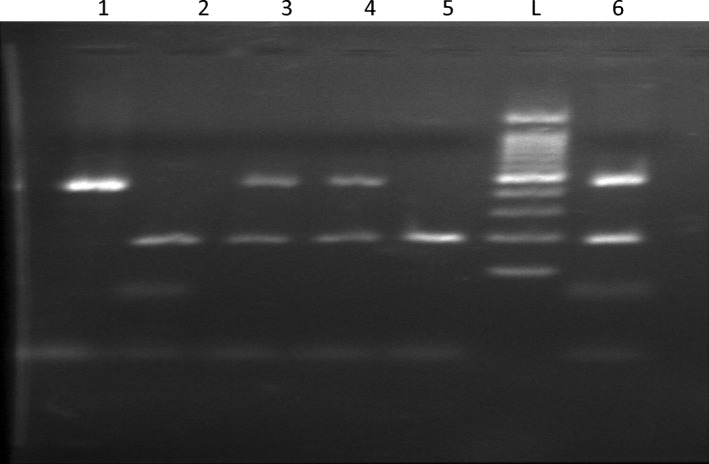
Agarose gel electrophoresis of ACE I/D gene Polymorphism; L represents ladder of 100 bp DNA, Lane 1 showed 490 bp (homozygous II), Lane 2,5 showed 190 bp (homozygous DD), Lane 3,4,6 showed 490 and 190 bp (heterozygous ID).

Extraction of DNA was performed from peripheral blood mononuclear cells through QIAamp® DNA Blood kit (Qiagen, Hilden, Germany) following the manufacturer's protocol. ACE gene (I/D) polymorphism was achieved according to Rigat et al.^19^ by conventional PCR with gel electrophoresis methods. In a reaction volume of 50 µL that contains10 pmoles of sense primer 5′ CTGGAGACCACTCCCATCC1TTCT 3′ and anti-sense primer 5′ GATGTGGCCATCACATTCGTCAGAT3′ and 1U of Taq polymerase, 0.5 mM of each dNTP (Promega), 3 mM MgCl2, 50 mM KCI, 10 mM Tris-HCI pH 8.4 and 50 ng/μl of genomic DNA. Through using PTC-100 thermal cycler, PCR program was set at 30 cycles (denaturation at 94 °C for 1 min, annealing at 58 °C for 1 min, and extension at 72 °C for 2 min. The PCR product was identified through agarose gel electrophoresis and ethidium bromide staining. A 190 bp fragment was shown in the absence of the insertion (D) and while a fragment of 490 bp was observed in the presence of the insertion (I). A third fragment, heterozygote one, a fragment at 190 bp and 490 bp corresponds to ID genotype was shown. The results were identified as DD, II and ID genotypes respectively as shown in Fig. 5.

### Statistical analysis

Data analysis was done through SPSS program version 18. Data was expressed as mean ± SD. Independent t-test was used to compare differences between groups. Significance was shown at P < 0.05. Pearson's correlation was done to study relation between variables. Mann–Whitney test was performed to compare variables which did not show normal distribution. One way analysis of variance (ANOVA) and a post hoc test of least significant differences (LSD) were used to test for differences between the DS and DNS and healthy control groups. Differences in genotype distribution between T2DM patients and controls groups were tested using Fisher’s exact test and χ2 test. ROC curve was constructed for detecting cutoff value of serum copeptin, KIM-1 and uPb showing optimum sensitivity and specificity in prediction of nephropathy among smoker diabetic patients.

### Ethics approval

Approval of the Ethical Committee in the National Research Centre, Egypt with number (13132032021) was taken before the study.

## Data Availability

The datasets generated or analysed during the present study could be available from the corresponding author upon reasonable request.
