# Author Correction: Potential role of serum copeptin among smoker T2DM patients with emphasis to ACE I/D gene polymorphism predicting DN

**DOI:** 10.1038/s41598-024-68545-x

**Published:** 2024-07-31

**Authors:** Mona Mohamed Taha, Mohamed Ahmed Yehia Zakaria, Yasmine Hamdy Eisa, Maysa Hatem Rashed

**Affiliations:** 1 Department of Environmental and Occupational Medicine, National Research Centre, Buhouth Street, Dokki, Cairo, Egypt; 2 Department of Forensic Medicine and Clinical Toxicology, Faculty of Medicine, October 6 University, Giza, Egypt; 3 Department of Public Health and Community Medicine, Faculty of Medicine, October 6 University, Giza, Egypt; 4 Department of Biochemistry and Molecular Biology, Faculty of Medicine, October 6 University, Giza, Egypt

Correction to: *Scientific Reports* 10.1038/s41598-024-62865-8, published online 06 June 2024

The original version of this Article contained errors in the spelling of the authors Yasmine Hamdy Eisa & Maysa Hatem Rashed, which were incorrectly given as Yasmine Hamed Eisa & Maisa Hatem Rashed respectively.

The original Article has been corrected.

